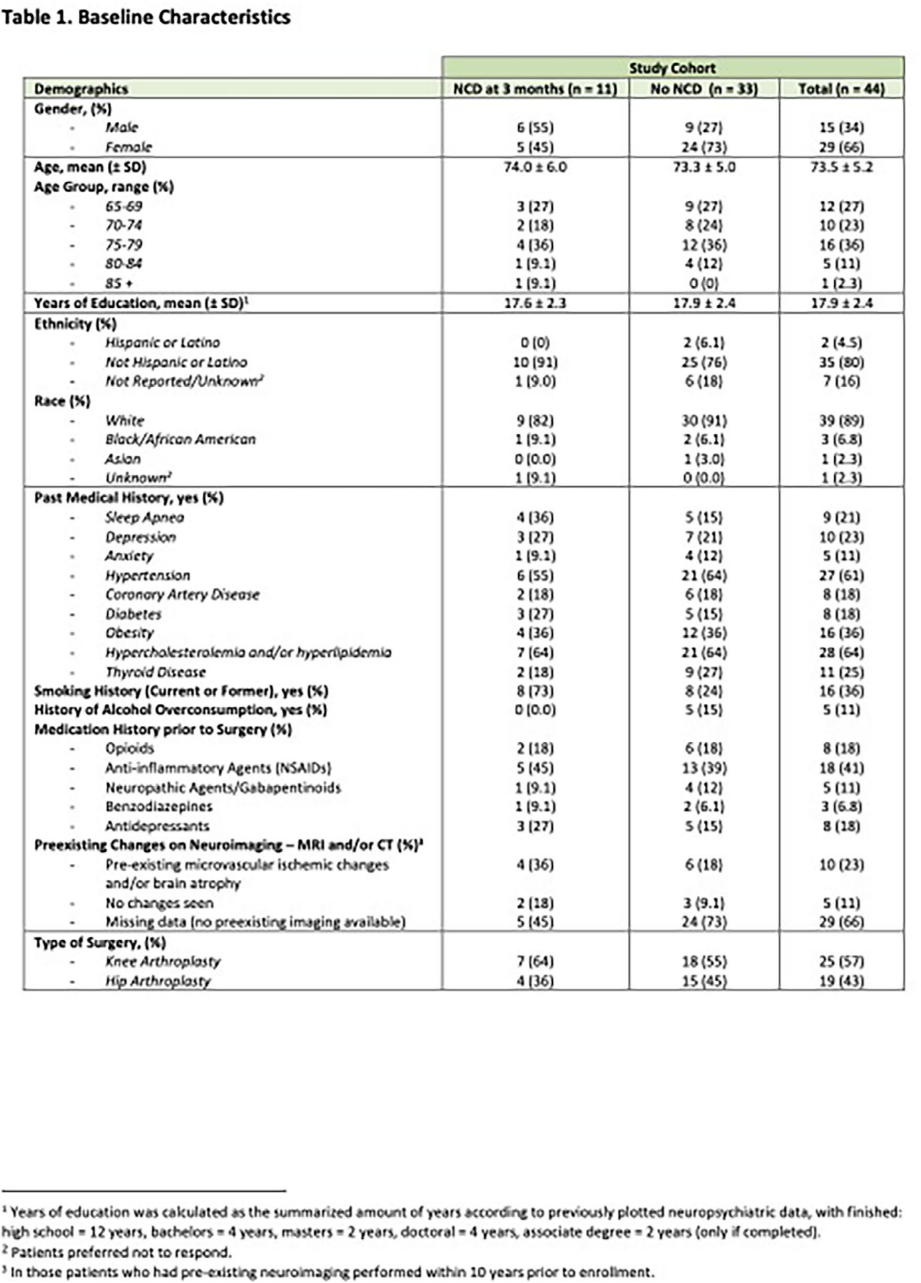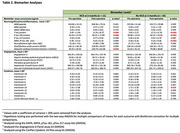# The Potential of Using ADRD‐related Biomarkers to Predict and Diagnose Perioperative Neurocognitive Disorders in Elderly Patients Undergoing Surgery

**DOI:** 10.1002/alz70856_101699

**Published:** 2025-12-24

**Authors:** Rachel R Wu, Elaine Zhu, Christina L Bi, Wonyoung Park, Raven Perez, Hamleini Martinez, Ekow B Commeh, Ludovic Debure, Romario B Denoon, Mobeena Ghuman, Braden V Saba, Wajiha Ahmed, Daniel Waren, Gabrielle Bruno, Courtney O'Brien, Vinay K Aggarwal, Joshua C Rozell, David Furgiuele, William Macaulay, Alok Vedvyas, Karyn Marsh, Ran Schwarzkopf, Evan T Schulze, Arjun V. Masurkar, Allal Boutajangout, Thomas Wisniewski, Ricardo S. Osorio, Lisa V Doan, Jing Wang, Mika Rockholt

**Affiliations:** ^1^ NYU Grossman School of Medicine, New York, NY, USA; ^2^ Center for Cognitive Neurology, New York University Langone Health, New York, NY, USA; ^3^ NYU Alzheimer's Disease Research Center, New York, NY, USA; ^4^ Center for Cognitive Neurology, New York, NY, USA; ^5^ NYU Langone Health, New York, NY, USA; ^6^ NYU Langone Medical Center, New York, NY, USA; ^7^ New York University Grossman School of Medicine, New York, NY, USA; ^8^ Alzheimer's Disease Research Center, New York University Langone Health, New York, NY, USA; ^9^ New York University Langone Health, New York, NY, USA; ^10^ NYU Langone School of Medicine/ Alzheimer's Disease Research Center, New York, NY, USA; ^11^ New York University Langone Health, New York University Grossman School of Medicine, New York, NY, USA; ^12^ NYU School of Medicine, New York, NY, USA; ^13^ Health Brain Aging and Sleep Center, New York, NY, USA; ^14^ Center for Sleep and Brain Health, Department of Psychiatry, NYU Langone Health, New York, NY, USA; ^15^ Nathan S. Kline Institute, Orangeburg, NY, USA

## Abstract

**Background:**

Research in the US increasingly focuses on the impact of surgery and anesthesia on an aging population, as 40% of surgical patients are adults aged 65 years and older. Evidence suggests a potent link between advancing age and an increased risk of neurocognitive disorders (NCD), influenced by neuroinflammation and blood‐brain barrier changes. Here, we analyzed changes in pre‐and post‐operative blood biomarkers and paired our findings with results from batteries of neurocognitive tests performed pre‐ and post‐surgery to evaluate changes in cognitive domains.

**Method:**

Patients aged 65 scheduled for hip or knee arthroplasty underwent evaluation with the UDS v3.0 T‐cog virtual assessment to establish baseline cognitive function prior to surgery. The T‐MoCA was used to determine baseline cognitive impairment (CI). Blood samples were drawn before surgery to establish a baseline, and after surgery to determine post‐surgical physiological changes. Cognitive assessments were administered 3 months post‐operatively, with a change in > –1 SD in Z‐scores in at least one cognitive domain defining NCD.

**Result:**

At the 3‐month post‐operative evaluation, 25% of patients (11/44) showed signs of NCD, with a higher prevalence in men (54% vs. 46%, *p* =  0.098). Age and education levels did not differ significantly between groups. Pre‐surgical biomarker levels did not differ significantly between groups, except in anti‐inflammatory IL‐4 levels which were lower in those who developed NCD. Both groups showed increases in T‐tau and *p*‐Tau 181, but only non‐NCD patients had a significant decrease in Aβ42/p‐Tau 181 ratio. Post‐operatively, people with NCD had significantly higher levels of UHCL‐1, while non‐NCD patients had higher anti‐inflammatory IL‐10. Among those with baseline CI and NCD, post‐surgery T‐tau levels were significantly higher (*p* = 0.030) compared to those without CI and NCD.

**Conclusion:**

Surgery may enhance Alzheimer's and related diseases biomarkers in both NCD and non‐NCD groups. The Aβ42/p‐Tau 181 ratio after surgery was significant in non‐NCD cases. NCD cases showed the same trend and an increase in sample size will likely show the same finding. Neuronal injury observed in NCD patients was confirmed by the elevation of UHCL‐1.